# Determinants of short and long term functional recovery after hospitalization for community-acquired pneumonia in the elderly: role of inflammatory markers

**DOI:** 10.1186/1471-2318-6-12

**Published:** 2006-08-09

**Authors:** Ali El Solh, Lilibeth Pineda, Pam Bouquin, Corey Mankowski

**Affiliations:** 1 Western New York Respiratory Research Center, 462 Grider Street, Buffalo, NY 14215, USA; 2 Department of Medicine, Division of Pulmonary, Critical Care, and Sleep Medicine, University at Buffalo School of Medicine and Biomedical Sciences, Buffalo, NY, USA

## Abstract

**Background:**

Hospitalization for older patients with community-acquired pneumonia (CAP) is associated with functional decline. Little is know about the relationship between inflammatory markers and determinants of functional status in this population. The aim of the study is to investigate the association between tumor necrosis factor (TNF)-α, C-reactive protein (CRP) and Activities of Daily Living, and to identify risk factors associated with one year mortality or hospital readmission.

**Methods:**

301 consecutive patients hospitalized for CAP (mean age 73.9 ± 5.3 years) in a University affiliated hospital over 18 month period were included. All patients were evaluated on admission to identify baseline demographic, microbiological, cognitive and functional characteristics. Serum levels for TNF-α and CRP were collected at the same time. Reassessment of functional status at discharge, and monthly thereafter till 3 months post discharge was obtained and compared with preadmission level to document loss or recovery of functionality. Outcome was assessed by the composite endpoint of hospital readmission or death from any cause up to one year post hospital discharge.

**Results:**

36% of patients developed functional decline at discharge and 11% had persistent functional impairment at 3 months. Serum TNF-α (odds ratio [OR] 1.12, 95% CI 1.08–1.15; p < 0.001) and the Charlson Index (OR = 1.39, 95% CI 1.14 to 1.71; p = 0.001) but not age, CRP, or cognitive status were independently associated with loss of functionality at the time of hospital discharge. Lack of recovery in functional status at 3 months was associated with impaired cognitive ability and preadmission comorbidities. In Cox regression analysis, persistent functional impairment at 3 months, impaired cognitive function, and the Charlson Index were highly predictive of one year hospital readmission or death.

**Conclusion:**

Serum TNF-α levels can be useful in determining patients at risk for functional impairment following hospitalization from CAP. Old patients with impaired cognitive function and preexisting comorbidities who exhibit delay in functional recovery at 3 months post discharge may be at high risk for hospital readmission and death. With the scarcity of resources, a future risk stratification system based on these findings might be proven helpful to target older patients who are likely to benefit from interventional strategies.

## Background

Pneumonia is one of the most common and significant health problems in the elderly. Together with influenza, it remains the eighth-leading cause in the United States and the leading infectious cause of death in this age group [1]. Approximately 366,000 elderly persons are discharged annually from short-stay hospitals after treatment for community-acquired pneumonia (CAP) [2] at a cost that exceeds $4.4 billion [3]. According to a recent analysis of the National Hospital Discharge Survey, the rates of hospitalization from pneumonia in this population have increased by 20 percent from 1988–1990 to 2000–2002 for patients aged 65 to 84 years old [4].

For elderly patients, hospitalization following an acute illness may lead to permanent functional declines or at times, even death [5]. Twenty five to 60% of older patients experience a loss of independent physical function while being treated in the hospital [6]. Recovery is usually prolonged especially in the frail elderly who may require up to several weeks to return to their baseline functional status. Cross sectional studies have identified significant association between increased inflammatory markers and functional disability [7,8]. Circulating levels of pro-inflammatory cytokines, such as interleukin (IL)-6 and tumor necrosis factor (TNF)-α, are usually elevated in CAP [9] while C-reactive protein (CRP) may correlate with severity [10]. However, the relationship of pro-inflammatory biomarkers to functional decline in older patients following an acute illness is unclear. After the implementation of a CAP clinical pathway, we conducted a prospective observational study to investigate the following aims: 1) to investigate the association between levels of TNF-α and CRP on admission and decrease in activities of daily living at the time of hospital discharge, 2) to determine the extent to which patients with various mortality risks recover to preadmission level of activities of daily living during the first 90 days after discharge, and 3) to identify risk factors associated with one year mortality or hospital readmission.

## Methods

### Study population

Between January 2003 and June 2004, all elderly patients (age ≥ 65 years old) admitted to a University-affiliated hospital with the diagnosis of CAP were considered for enrollment. Community-acquired pneumonia was defined according to the following criteria: 1) the presence of a new pulmonary infiltrate on the chest radiograph; 2) the new onset of at least two of the following symptoms: cough, dyspnea, chest pain, change in mental status; 3) temperature ≥38°C or ≤36.0°C, and/or leukocytosis (>11.0 × 10^9^/L) or neutropenia (3.5 × 10^9^/L); and 4) the absence of evidence of a cause other than pneumonia. Patients residing in nursing homes or who were hospitalized in the last 90 days were not included. Patients with aspiration, severe immunosuppression (solid organ transplantation, steroid therapy ≥10 mg/d for more than 2 weeks, known Human Immunodeficiency Virus, or Acquired Immunodeficiency Syndrome defining criteria), underlying active malignancy, or who had a Do-Not Resuscitate order (DNR) were also excluded. The Institutional Review Board for human studies approved the study. Participants or their family members gave informed consent at the time of enrollment.

### Pneumonia pathway

An admission pneumonia pathway was developed by the hospital committee for quality improvement for the management of CAP. The pathway included preprinted orders that included assessment of oxygen saturation, collection of two sets of blood cultures, choice of antimicrobial therapy between a third generation cephalosporin plus a macrolide or a fluoroquinolone intravenously, and administration of pneumococcal and influenza vaccines if no contraindication exists. Counseling in the form of literature review or a video taped seminar regarding smoking cessation was provided during hospital admission. Once clinical stability was achieved (defined as heart rate ≤100 beat/min, systolic blood pressure ≥90 mmHg, oral temperature ≤37.5°C for 24 hour, and return of oxygen saturation to baseline at ≥90%) [11], an assessment of safe discharge was determined based on functional status and social support.

### Data collection

Enrolled patients were assessed by a member of the research team within 24 hours of hospital admission. Records were examined carefully and data on demographic information, and clinical presentation was gathered. The Pneumonia Severity Index (PSI) score [12] and the Charlson Comorbidity Index [13] were obtained at the time of admission. Patients or their next of kin were asked to report on their ability to perform six activities of daily living (bathing, dressing, transferring, walking, toileting, and eating) two weeks prior to admission. For each area of activity, patients were assigned a score of ranging from 1 if they were fully independent to 3 if they were completely dependent. An Activity of Daily Living (ADL) score [14] was generated. An abbreviated Mini-Mental State Examination (MMSE) was also obtained during the admission interview [15]. The abbreviated MMSE omits the language items of the test: naming, repetition, three-stage command, reading, writing, and copying because of concern about the ability of acutely ill elderly patients to complete these tasks.

All chest roentgenograms were evaluated independently by two pulmonologists (AES and LP). Differences among the two examiners were resolved by consensus. Data on antimicrobial coverage, time to administration, and total duration were recorded.

### Microbiology

Regular sampling was done of sputum, blood cultures and serum for serology. Sputum was Gram stained. Representative sputum originating from the lower respiratory tract was defined as that containing > 25 granulocytes and < 10 epithelial cells per low power field (total magnification × 100) [16]. Sputum and pleural fluid were cultured in the following medium: 5% sheep's blood, chocolate agar, and MacConkey plates. The etiology of pneumonia was considered as definitive under the following conditions: 1) isolation of a pathogen in cultures of blood or pleural fluid; 2) increase of IgM titers for *Chlamydia pneumoniae *(IgM > 1:32), *Coxiella burnetii *(IgM > 1:80), *Mycoplasma pneumoniae *(any positive titer); 3) positive direct fluorescent antibody for influenza virus; 4) a single titer (IgG >1:128) or a positive urinary antigen for *Legionella pneumophila*; or 5) a positive urinary antigen for *Streptococcus pneumoniae *[17,18]. Valid samples of sputum growing a predominant microorganism were considered for a probable bacteriological diagnosis [17].

### Outcome

We defined a decline in functional status *a priori *as a 2-point increase on the ADL scale. Conversely, a 2-point decrease on the ADL scale was interpreted as an improvement in functional status. All patients received a standard follow up call at 30, 60, and 90 days after discharge to ascertain functional status recovery. All follow up interviews were conducted by the same personnel. Outcome was assessed by the composite endpoint of hospital readmission or death from any cause up to one year post hospital discharge. An unfavorable outcome was defined as hospital readmission or death. Vital records from the New York Department of Public Health (regarding in-state deaths) and the Social Security Administration Death Master File [19,20] (regarding out-of-state deaths) were used to determine or confirm death at one year post discharge. Readmissions were determined from participants, family reports, or medical records.

### Systemic markers of inflammation

Venous blood samples were collected from all participants at the time of admission to the general wards. Specimens were placed immediately on ice and rapidly transferred to the laboratory to be centrifuged and stored at -70°C. Assays for CRP and TNF-α levels were performed in a single batch by one of the authors who was blinded to the clinical details of individual patients. Plasma TNF-α levels were measured in duplicate using enzyme-linked immunosorbent assay (ELISA) kits (R & D Systems, Minneapolis, MN). The detectable limit for TNF-α was 0.18 pg/mL. Plasma levels of CRP were also measured in duplicate using ELISA with anti-CRP antibodies (Calbiochem, San Diego, CA). The CRP assay was standardized and had a sensitivity of 0.08 mg/mL [21].

### Statistical analysis

The results are presented as mean ± SD or median with interquartile range (IQ). Continuous variables were analyzed using a two-tailed student's *t *test for normally distributed variables; otherwise difference in median values was assessed using nonparametric tests (Mann-Withney *U *test for two groups of continuous data and the Kruskal-Wallis test for three or more groups). For categorical variables, testing for difference in proportions was performed using the Chi-square or the Fisher's exact test when indicated. Pairwise correlation was performed using the Spearman rank correlation coefficient. Missing values for PSI scores were encountered in less than 4% of the total population sample. Multiple imputations were used to assess missing values [22]. Factors with p < 0.10 in our univariate analysis were entered into a multiple logistic regression model. All potential explanatory variables included in the multivariable analyses were subjected to correlation matrix for analysis of collinearity. Variables with association among each other were not included in the multivariable model. Interactions were explored between the variables retained in the multivariable models. Cumulative-event curves were estimated with the Kaplan-Meier method, and groups compared using the log-rank test. A multivariate analysis of time to composite end point was performed using Cox proportional hazards regression model. Statistical significance was defined as p < 0.05. Analyses were performed using StatView 5.0 (SAS Institute Inc., Cary, NC) and SPSS 12.0 (SPSS Inc., Chicago, IL) software.

## Results

### Characteristics of the study population

**Figure 1:**
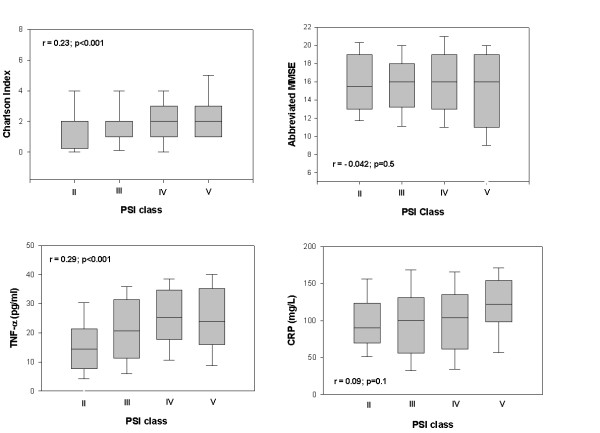
Distribution of the Pneumonia Severity Index scores, theCharlson Index, the abbreviated MMSE, serum TNF-α, and CRP according to PSI classes. Data are provided in box plot format (median, 25%–75%).

Three hundred one consecutive patients (mean age 73.9 ± 5.3 years) participated in the study. Sixty percent of the participants were male. Thirty six percent had one major comorbid illness and 51% had two or more. Chronic pulmonary diseases (33%), diabetes mellitus (20%), and congestive heart failure (10%) accounted for the majority of chronic diseases. According to the PSI score on admission, 45% of patients were low-risk cases (class II-III), 41% were moderate risk (class IV), and 14%, high risk (class V). The median age of patients in each class was 71 years (range 65 to 77) for class II, 72 years (range 65 to 89) for class III, 74 years (range 67 to 91) for class IV, and 75 years (range 69 to 94) for class V. Thirty nine percent had an abbreviated MMSE of 14 or lower while 61% scored 15 or higher. Figure 1 displays the baseline PSI scores, Charlson Index, abbreviated MMSE, serum TNF-α, and CRP levels in function of PSI class categories. A significant correlation was observed between PSI class severity and each of the following variables: TNF-α (R = 0.29, 95% confidence interval (CI) 0.18 to 0.4; p < 0.001) and Charlson Index R = 0.23, 95% CI 0.12 to 0.34; p < 0.001) but not with CRP (R = 0.09, 95% CI-0.02 to 0.20; p = 0.11) or abbreviated MMSE (R = -0.042, 95% CI-0.15 to 0.07; p = 0.46).

The composite end point of hospital readmission or death was reached in 62 (21%) patients over the course of a one year period. Thirteen patients were lost for follow up after discharge. Thirty three patients were readmitted to a hospital with a median time from discharge of 176 days (range 17–341 days). None of the readmissions was considered an elective admission. The reasons for readmission included recurrence of pneumonia (n = 6), cardiovascular events (n = 9), exacerbation of chronic pulmonary disease (n = 11), and other acute conditions (n = 7). Case fatality rate was 10% at 12 months. The causes of death reported were pneumonia (n = 4), ischemic heart disease (n = 11), acute cerebrovascular accident (n = 5), and chronic pulmonary obstructive disease (n = 7). In two cases, the cause of death was not known.

Table 1 depicts the demographic characteristics of those with favorable and unfavorable outcome. The two groups were comparable in age, gender distribution, and smoking history. However, a higher Charlson Index and a lower cognitive and functional status were observed in those with unfavorable outcome (p = 0.002, p = 0.006, and p = 0.01; respectively). The clinical presentation and radiographic patterns of the study population are shown in table 2. Although those with unfavorable outcome had a higher PSI score, the distribution of patients by risk of mortality was not significantly different between the two groups. TNF-α and CRP levels were also comparable between those with favorable and unfavorable outcomes.

**Table 1:** Demographics characteristics of the study population

	Favorable outcome (n = 239)	Unfavorable outcome (n = 62)	P value
Age, mean, *years*	72.6 ± 8.3	74.4 ± 6.7	0.08
Gender (Male/Female), *n*	144/95	36/26	0.8
Abbreviated MMSE	15.9 ± 3.3	14.2 ± 4.4	0.006
Preadmission ADL	7.7 ± 2.7	8.9 ± 3.4	0.01
			
**Comorbidities**, *n (%)*			
Congestive heart failure	23 (10)	7 (11)	0.9
Chronic pulmonary diseases	76 (32)	23 (37)	0.5
Cerebrovascular accident	15 (6)	6 (10)	0.5
Diabetes mellitus	43 (18)	16 (26)	0.2
**Charlson Index, ***n (%)*			0.002
0	36 (15)	3 (5)	
1–2	147 (62)	33 (53)	
3–4	51 (21)	20 (32)	
≥5	5 (2)	6 (10)	
**Smoking status, ***n (%)*			
Currently smoking	21 (9)	9 (15)	0.3
Past smokers	82 (34)	25 (40)	0.4

**Table 2:** Clinical characteristics of the study population

	Favorable outcome (n = 239)	Unfavorable outcome (n = 62)	P value
**Symptoms, ***n (%)*			
Cough	153 (64)	37 (60)	0.6
Dyspnea	160 (67)	43 (69)	0.8
Chest pain	29 (12)	10 (16)	0.5
**PSI score, **mean ± SD	93.5 ± 27.7	104.3 ± 32.2	0.02
**PSI Classes, ***n (%)*			0.1
Class II	47 (20)	10 (16)	
Class III	69 (29)	10 (16)	
Class IV	92 (38)	30 (49)	
Class V	31 (13)	12 (19)	
			
**Chest radiographs, ***n (%)*			
Multilobar disease	45 (19)	16 (26)	0.3
Bilateral disease	35 (15)	11 (18)	0.7
Pleural effusion	20 (8)	9 (15)	0.2
			
Inflammatory markers*			
TNF-α, pg/ml	23.5 (15.4–31.3)	25.9 (16.9–3.8)	0.3
CRP, mg/L	101.4 (64.4–124.3)	113.2 (77.8–140.1)	0.1

*Expressed as median with interquartile range

Two hundred seventeen (72%) participants received a fluoroquinolone and 84 (28%) had a combination of a third generation cephalosporin plus a macrolide. A fluoroquinolone was prescribed for 171 of patients with favorable outcome compared to 46 for those with unfavorable outcome (p = 0.8). The median time to initial hospital antimicrobial therapy was comparable at one hour post presentation to the emergency department for both groups (range 1–3 hours; p = 0.9). Similarly, the duration of hospitalization was not significantly different (6.8 ± 5.2 days for those in the favorable outcome and 7.5 ± 5.3 days for those in the unfavorable outcome group, respectively; p = 0.35). Home care services were set up for 22 patients (13 and 9 for the favorable and unfavorable outcome group, respectively) while discharge to a nursing home occurred in 8 (5 and 3, respectively). None of the participants underwent rehabilitation during the period of the study.

### Microbiology

Two serial blood cultures (n = 292, 97%) and serology (n = 253, 84%, acute illness single sample) were performed. A sputum sample was obtained in 235 (78%) patients but culture was only performed in good quality samples (n = 141, 47%). Detection of the *L. pneumophila *(serotype 1) urinary antigen by ELISA was performed in 265 (88%) cases. Pleural fluid culture was carried out in 13 (4%) cases.

Overall, 139 pathogens were detected, of which 82 (59%) were detected by sputum examination and 57 (41%) were detected by other techniques. *S. pneumoniae *constituted 47 of the 139 pathogens (34%), and was the most frequent pathogen. Other pathogens included *Hemophilus influenzae *(n = 25, 18%), *Chlamydia pneumoniae *(n = 20, 14%), *Legionella pneumophila *(n = 14, 10%), and *Moraxella catarrhalis *(n = 9, 6%), respectively. The highest values for TNF-α and CRP levels were observed in patients with pneumonia caused by *Streptococcus pneumoniae *and *Legionella pneumophila *(table 3). However, there were no differences in serum TNF-α or CRP values when the different etiologic groups were compared. Of interest, median TNF-α but not CRP levels were significantly higher in patients with microbiologically-confirmed pneumonia (23.4 pg/ml IQ [17.9–31.5] and 114.1 mg/l IQ [71.4–144.9]; respectively) compared to those with microbiologically-unconfirmed pneumonia (20.2 pg/ml IQ [10.8–32.3] and 102.9 IQ [59.7–133.8]; respectively) (p = 0.002 and 0.08; respectively).

**Table 3:** Microbial etiology and corresponding TNF-α and CRP levels

Microorganisms	Definite (n)	Probable (n)	TNF-α (pg/ml)*	CRP (mg/L)*
*Streptococcus pneumoniae*	16	36	49.8 (36.6–76.1)	127.3 (111.7–160.3)
*Mycoplasma pneumoniae*	2	-	16.9 (12.9–22.6)	56.6 (41.7–118.9)
*Moraxella catarrhalis*	-	9	19.8 (14.7–26.0)	85.2 (55.0–123.9)
*Chlamydia pneumoniae*	20	-	21.1 (15.4–28.3)	98.7 (61.6–132.3)
*Hemophilus influenzae*	-	25	23.2 (17.8–35.2)	103.5 (67.2–123.1)
*Klebsiella pneumonia*	1	7	28.7 (17.5–36.6)	122.4 (115.9–128.9)
*Escherichia coli*	-	5	22.1 (18.4–25.9)	101.8 (64.7–145.7)
*Legionella pneumophila*	14	-	32.9 (26.5–55.1)	127.5 (106.8–176.3)
Influenza virus	4	-	18.2 (13.2–23.3)	76.6 (43.8–116.7)

*Expressed as median with interquartile range

### Risk factors for functional decline on discharge

**Figure 2:**
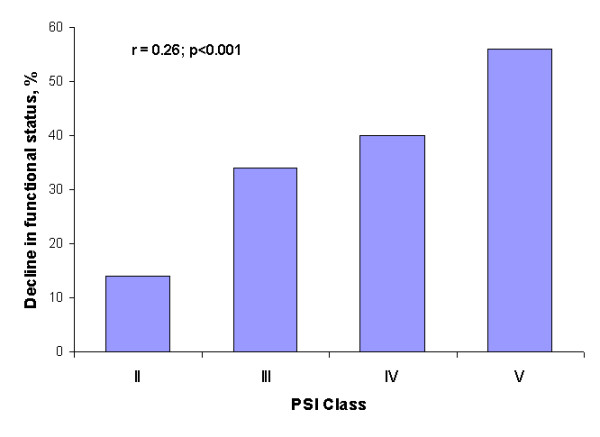
Frequency plot of decline in functional status stratified by the Pneumonia Severity Index classes (p < 0.001).

A total of 108 (36%) out of 301 participants had a decline in functional status on hospital discharge. As shown in figure 2, higher risk categories were associated with higher rates of functional decline at discharge (R = 0.26, 95% CI 0.15–0.36; p < 0.001). A significant association was also detected between functional decline and serum TNF-α levels (R = 0.46, 95% CI 0.36–0.54; p < 0.001), Charlson Index (R = 0.24, 95% CI 0.14–0.35; p < 0.001), abbreviated MMSE (R = -0.15. 95% CI-0.26 to -0.037; p = 0.01) and length of hospital stay (R = 0.27, 95% CI 0.17–0.38; p < 0.001) but not with age or CRP levels.

In multivariate analysis, only serum TNF-α (odds ratio [OR] 1.12, 95% CI 1.08–1.15; p < 0.001) and the Charlson Index (OR = 1.39, 95% CI 1.14 to 1.71; p = 0.001) were independently associated with decline in functional status at the time of hospital discharge.

### Functional recovery

**Figure 3:**
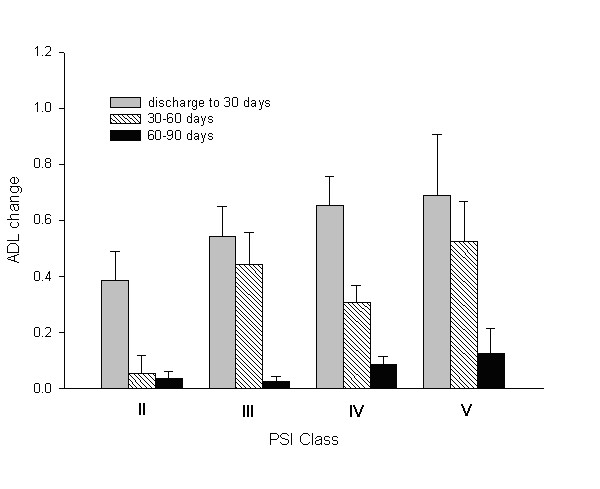
Change in Activity of Daily Living score (mean ± SD) for each of the Pneumonia Severity Index class at each successive follow-up period.

The change in ADL score at each successive follow-up period is presented in figure 3. For the period ranging from hospital discharge up to 30 days, the improvement in ADL was the largest in all four risk categories. The improvement was correlated with the PSI risk severity (p = 0.03) as the largest change in ADL was noted in Class V. From days 30 to 60, the absolute change in ADL was the smallest in Class II but an improvement in function status was still observed in the remaining risk classes. In comparison, the third follow up period (60 to 90 days) showed the smallest amount of change in terms of ADL improvement across all classes. At the end of the follow-up period of 90 days, 34 (12%) patients out of 290 remaining showed persistent decline in functional status compared to preadmission. In multivariate analysis, persistent decline of functional status after 90 days of hospital discharge was associated with abbreviated MMSE (OR 0.86; 95% CI 0.79–0.94; p = 0.001) and preadmission Charlson Index (OR 1.28; 95% CI 1.04–1.59; p = 0.02) but not age, PSI score, TNF-α, CRP levels, or microbial etiology.

### Predictors of outcome

**Figure 4:**
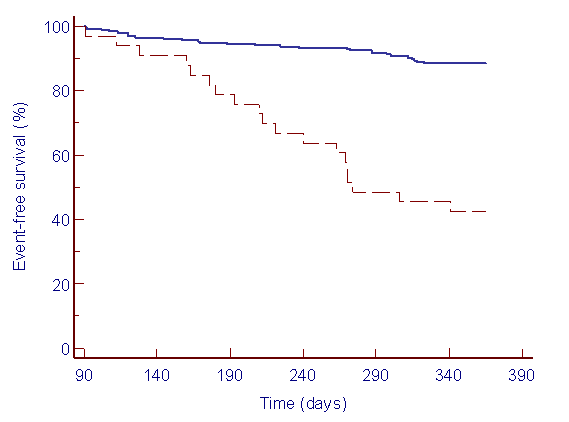
Kaplan-Meier plot of one year hospital readmission or death by functional decline at day 90 post hospital discharge from CAP. Persistent functional decline at day 90 (broken line) versus recovery of functional status by day 90 (continuous line) (logrank test p < 0.001).

The lack of improvement in ADL was observed more frequently in those with unfavorable outcome at all time points (table 4). The probability of event-free survival was significantly lower for patients who showed persistent lack of functional recovery at day 90 post hospital discharge (p < 0.001) than those who did not (figure 4). In a Cox regression analysis, lack of recovery in functional status at day 90 post discharge (Hazard Ratio (HR) 6.16, 95%CI 3.42–11.07; p < 0.001), abbreviated MMSE (HR 0.91; 95% CI 0.84–0.98), and the Charlson Index (HR 1.24, 95% CI 1.03–1.51; p = 0.025) were highly correlated with hospital readmission or death. Neither age, TNF-α, CRP nor PSI score was significantly associated with unfavorable outcome.

**Table 4:** Comparison of functional status at follow up time points to preadmission status

**Functional status (ADL)**	Favorable outcome (n = 239)	Unfavorable outcome (n = 62)	P value
**Compared to preadmission**			
Decline on discharge,*n (%)*	69 (29)	39 (63)	<0.001
Decline at day 30, *n (%)*	39 (16)	36 (58)	<0.001
Decline at day 60, *n (%)*	17 (7)	24 (39)	<0.001
Decline at day 90, *n (%)*	14 (6)	20 (32)	<0.001

## Discussion

The physiologic changes associated with aging predispose older patients to serious complications following hospitalization. In the current study, 36% and 11% of the patients experienced functional impairment following hospitalization for CAP and at 3 months post discharge. These figures are consistent with those reported in the Hospital Outcomes Project for the Elderly study which showed that 1 in 3 elderly patients developed decline in functional status after being hospitalized for an acute illness and about out 1 in 5 failed to recover after 3 months [23]. Several risk factors of functional decline in older persons have been described previously following acute illness including advanced age, previous impaired functional status, and lower scores on the abbreviated MMSE [15,24]. Other authors have found the presence of bedsores and hypoalbuminemia as independent factors of decline in ADL at the time of discharge but age and comorbid diseases were not [25]. Our study expanded those findings by showing a positive correlation between serum TNF-α levels and functional decline irrespective of demographic and clinical characteristics. The higher the TNF-α levels were on admission, the higher was the risk of decline in ADL. Torres and colleagues [26] identified a similar relationship between PSI and functional decline in hospitalized elderly patients with pneumonia. These associations suggest that the severity of infection affected function in a dose-dependent response. Because TNF-α can act directly on mature muscle to accelerate protein degradation [27], the loss of muscle mass following hospitalization for CAP may contribute to significant weakness and loss of mobility in the elderly which in turn would result in functional decline.

Our data indicated a similar trend between CRP levels and decline in ADL on discharge but the correlation was weaker and did not reach statistical significance. The poor association between functional status and CRP is highlighted by the conflicting observations in previously published series where the relationship between CRP levels and severity of illness in CAP has not been consistent [28,29]. Seppa and colleagues [30] reported that CRP level ≥100 mg/L was a marker independently associated with higher risk of death in patients with lower respiratory tract infections. Additionally Hedlund [31] noted that those patients with higher CRP levels had longer duration of fever and hospital stay and had delayed clinical and radiographic recovery on follow up after 8 weeks of discharge. Yet, Vazquez and colleagues [28] found that CRP levels were not correlated with PSI score. Although the CRP levels in our study were higher in those with microbiologically established CAP, CRP is a non-specific marker of inflammation and is subject to the influence of age, gender, and comorbid conditions that may act as an important stimulus for its hepatic synthesis.

Prior studies have pointed to an association between increased length of hospital stay and decline in functional status at the time of discharge [23,32]. In our cohort, 45% of patients who remained hospitalized for more than 7 days developed functional decline compared to 32% who were discharged within 7 days. Furthermore, the functional decline continued further till 3 months after hospital discharge. However, in multivariate analysis, this association lost its significance. The length of stay in our model acts as a proxy for severity of illness and reflects the consequences of deconditioning effect of bed rest and iatrogenic complications [33].

In line with other investigations [34,35], we found that the vast majority of newly disabled community-dwelling older persons recovered independent function at day 90 post hospital discharge. Persons who were cognitively impaired or severely burden with coexisting diseases were less likely to recover and more likely to be hospitalized or to succumb to their underlying diseases within one year of hospital discharge for CAP. The Hospital Admission Risk Profile Index (HARP) [15] that was developed to identify older patients at risk for functional decline following acute medical illness incorporated similar characteristics to those found significant in our model: ADL and abbreviated MMSE. Unlike the HARP study, age was not an independent factor in our study but the burden of comorbidities was. With the scarcity of resources, a future risk stratification system based on these findings might be proven helpful to target older patients who are likely to benefit from interventional strategies.

Strengths of our study include the prospective design, the homogeneity of our study population, the high reliability and accuracy of prognostic indices, and the low rate of attrition. While other studies [25,34] have addressed the question of functional decline and recovery in the elderly following an acute illness, we have limited our investigation to older patients with CAP after implementing an approved CAP pathway. Because the nature of the underlying acute illness may impact the rate of recovery independently [36], our findings would be more relevant when evaluating long term management of older patients with CAP. The present study also has its limitations that warrant further discussion. First, the reliance on proxies' report of ADL function at the time of admission as well during the time of telephone interviews may have resulted in inaccurate estimation of functional declines because of the biases documented in other studies comparing caregiver reports of patient health with self-report information [37,38]. However it is unlikely that this bias seriously affected results because previous studies have shown that surrogate bias is lowest for directly observable measures [39]. Furthermore, excluding these patients would have introduced selection bias that would have favored those with less disease severity. Second, the acute illness may have affected our baseline measurement of cognitive function. Unfortunately, no true criterion standard exists for determining preexisting cognitive dysfunction [40]. Third, the rate of readmission and death might have been underestimated if participants have moved out of state or adopted different identification numbers. Fourth, our study population was limited to a single hospital. Initial processes of care and microbiological variation might not be reproducible in other geographic areas and could limit the generalization of our findings. However, our population did reflect the demographic characteristics of persons aged 65 years or older in Western New York, which are comparable to the United States as a whole. Fifth, we have not included an assessment of the socioeconomic status of our study population. There have been conflicting results concerning the relationship between socioeconomic status and pneumonia in terms of both diagnosis and outcome. Three studies [41-43] have reported an increased relative risk for lower social class quintiles and pneumonia and bronchitis mortality. Other studies, looking at pneumonia diagnosis and SES found no relation between the two [44,45]. We should point out that we have not assessed the processes of post hospital care that may have contributed to prolonged functional disability in susceptible patients. This factor should be taken into consideration when interpreting the results of our study.

## Conclusion

Based on the results of this study, older persons hospitalized for CAP may be at increased risk of functional loss both at the time of, and after discharge. TNF-α could be partially responsible for the severity of functional decline and may be useful to identify patients at risk. Functional recovery may be correlated with PSI class severity. Absence of functional improvement after 90 days of hospital discharge appears to be a strong predictor of hospital readmission and mortality. Further research is needed on the complex interaction between biological markers and recovery of functional status.

## Abbreviations

TNF-α Tumor necrosis factor

CRP C-reactive protein

CAP Community-acquired pneumonia

PSI Pneumonia Severity Index

ADL Activity of Daily Living

MMSE Mini mental status examination

HARP Hospital Admission Risk Profile Index

## Competing interests

The author(s) declare that they have no competing interests.

## Authors' contributions

AES conceived of the study, assisted in patient recruitment, drafted, and edited the manuscript.

LP participated in the design and recruitment of participants. PB performed serum TNF-α and CRP analysis. CM collected baseline information and conducted all follow-up interviews

All authors read and approved the final manuscript.

## Pre-publication history

The pre-publication history for this paper can be accessed here:


